# Immunomodulatory Effect of Bisphosphonate Risedronate Sodium on CD163^**+**^ Arginase 1^**+**^ M2 Macrophages: The Development of a Possible Supportive Therapy for Angiosarcoma

**DOI:** 10.1155/2013/325412

**Published:** 2014-02-16

**Authors:** Taku Fujimura, Yumi Kambayashi, Sadanori Furudate, Aya Kakizaki, Setsuya Aiba

**Affiliations:** Department of Dermatology, Tohoku University Graduate School of Medicine, Seiryo-machi 1-1, Aoba-ku, Sendai 980-8574, Japan

## Abstract

An imbalance of immunosuppressive cells and cytotoxic cells plays an important role in inhibiting the antitumor immune response of the tumor-bearing host. We previously reported the profiles of tumor infiltrating leukocytes in cutaneous angiosarcoma (AS) and suggested that a combination of docetaxel (DTX) with bisphosphonate risedronate sodium (RS) might be effective for MMP9-expressing AS by targeting immunosuppressive cells such as M2 macrophages. To further confirm the effect of this combination therapy, in this report we investigated the immunomodulatory effect of DTX and RS on CD163^+^ arginase 1 (Arg1)^+^ M2 macrophages in vitro. Interestingly, our present study demonstrated that DTX in combination with RS significantly upregulated the mRNA expression of CXCL10 on M2 macrophages and significantly decreased the mRNA expression of CCL17 and Arg1. Moreover, the production of CXCL10 and CXCL11 from M2 macrophages was significantly increased by DTX with RS though there was no effect of DTX with RS on the production of CCL5 and CCL17. Furthermore, DTX with RS significantly decreased the production of CCL18, which was previously reported to correlate with the severity and prognosis in cancer patients. Our present report suggests one of the possible mechanisms of DTX with RS in the supportive therapy for angiosarcoma.

## 1. Introduction

Angiosarcoma (AS) is a rare but highly aggressive vascular tumor that spreads widely throughout the skin, recurring locally and metastasizing early [1]. Standard treatment with resection and adjuvant radiotherapy results in local control in approximately 50% of patients at 1 year and a median survival of approximately 8 months [1–3]. Therefore, further adjuvant or supportive therapy is necessary for the treatment of AS. We previously reported that approximately 80% of AS expressed MMP9 [4] and that MMP9 might be another target for supportive therapy by using docetaxel (DTX) and bisphosphonate risedronate sodium (RS) for AS [4, 5]. Interestingly, our previous report also described the dense infiltration of CD163^+^ M2 macrophages and MMP9-bearing macrophage-like cells into the tumor, suggesting a contribution of M2 macrophages to the pathogenesis of AS [5]. Therefore, we hypothesized that one target for our supportive therapy might be these tumor-associated M2 macrophages. To further confirm the effect of our supportive therapy, in this report we investigated the immunomodulatory effect of DTX and RS on CD163^+^ arginase 1 (Arg1)^+^ M2 macrophages in vitro.

## 2. Material and Methods

### 2.1. Reagents

We used the following antibodies (Abs) for immunohistochemical staining: mouse monoclonal Abs for human CD163 (Novocastra, UK) and rabbit polyclonal Abs for human MMP9 (Abcam, Japan). We used the following antibodies (Abs) for flow cytometry: PE-conjugated anti-CD163 (R&D system, Minneapolis, MN, USA), PE-conjugated isotype control Ab (BD Bioscience), FITC-conjugated anti-arginase 1 (R&D system), or FITC-conjugated isotype control Ab (R&D system). We also used the following agents for the cell culture: recombinant human M-CSF and recombinant human IL-4 purchased from Peprotech (London, UK). Doxetaxel and bisphosphonate risedronate sodium were purchased from Wako Pure Chemical Industries (Osaka, Japan). 

### 2.2. Tissue Samples and Immunohistochemical Staining

We collected archival formalin-fixed paraffin-embedded skin specimens from patients with cutaneous angiosarcoma (AS) treated in the Department of Dermatology at Tohoku University Graduate School of Medicine. We defined AS by the typical clinical features and histological characteristics such as irregular anastomosing vascular channels lined by a single layer of enlarged endothelial cells lying between collagen bundles and by the immunohistochemical profiles (positive for CD31 and CD34). Cutaneous AS samples were processed for single staining of CD163 and MMP9 and developed with liquid permanent red (Dako A/S, Glostrop, Denmark) as described previously [6]. 

### 2.3. Culture of M2 Macrophages from Human Peripheral Blood Monocytes and Treatment with or without DTX and RS

CD14^+^ cells were isolated from peripheral blood mononuclear cells (PBMCs) from healthy donors using MACS beads (CD14 microbeads, Miltenyi Biotec Inc., Sunnyvale, CA, USA) according to the manufacturer's protocol. Briefly, after several washes with PBS, 1 × 10^8^ PBMCs were treated with 150 mL of the anti-human CD14 MACS beads in 600 mL of PBS supplemented with 1% bovine serum albumin (BSA, Invitrogen Japan K.K., Tokyo, Japan) and 5 mM EDTA (MACS buffer) at 4°C for 30 min. After washing with MACS buffer, the cells coated with CD14 microbeads were separated by a magnetic cell separator, MACS (Miltenyi Biotech), according to the manufacturer's protocol. This procedure yielded predominantly CD14^+^ cells with purity greater than 98% as assessed by FACS analysis. CD14^+^ monocytes (2 × 10^5^/mL) were cultured in complete medium containing 100 ng/mL of recombinant human M-CSF for 5 days. On the fifth day, monocyte-derived M2 macrophages (M2Mf) were treated with recombinant human IL-4 (20 ng/mL), after which we added DTX (1 *μ*g/mL) with or without RS (10 *μ*g/mL). This study was approved by the ethics committee of Tohoku University Graduate School of Medicine, Sendai, Japan.

### 2.4. Flow Cytometry

The surface expression of CD163 and intracellular expression of arginase 1 of M2M*ϕ* were analyzed by flow cytometry. For detecting the cytoplasmic cytokine expression, we used Golgi plug (BD Bioscience) 4 hours before cell harvest. We fixed and permeabilized the cells using Cytofix/Cytoperm solution (BD Bioscience) according to the manufacturer's protocol, and then cell staining was conducted using a combination of PE-conjugated anti-CD163 (10 *μ*g/mL) or PE-conjugated isotype control (10 *μ*g/mL) Ab and FITC-conjugated anti-arginase 1 or FITC-conjugated isotype control (10 *μ*g/mL) Ab. After washing with PBS supplemented with 1% BSA and 0.02% NaN3 (FACS buffer), the cells were analyzed by a C6 flow cytometer (Acuri Cytometers Inc., Ann Arbor, MI, USA).

### 2.5. RNA Extraction, Assessment of Quality and Reverse Transcription

Total RNA was extracted using an RNeasy Micro kit (Qiagen, Courtaboeuf, France) in accordance with the manufacturer's instructions. The RNA was eluted with 14 *μ*L of RNase-free water. DNase I treatment (RNase-Free DNase Set; Qiagen Courtaboeuf, France) was performed to remove contaminating genomic DNA. Reverse transcription was carried out with the SuperScript VILO cDNA Synthesis Kit (Invitrogen).

### 2.6. Quantitative Real-Time PCR Experiments

Amplification reactions were performed using a Mx 3000P Real-Time Quantitative PCR System (Stratagene). The thermal cycling condition were 3 min for polymerase activation at 95°C and 60 cycles at 95°C for 5 s and 60°C for 20 s and, finally, at 4°C. Relative mRNA expression levels were calculated for each gene and each time point after normalization against GAPDH, using the ΔΔCt method. 

### 2.7. Cytokine ELISA

After the 48-hour stimulation, supernatants were collected and the secretion of CCL5 (R&D systems), CCL17 (R&D systems), CCL18 (R&D systems), CCL22 (R&D systems), CXCL10 (R&D systems), CXCL11 (R&D systems), IFN-*γ* (R&D systems) and IL-10 (BD Bioscience) was determined by ELISA according to the manufacturer's instructions. Briefly, cytokines from the culture supernatants were immobilized onto 96-well plates and detected by the respective biotinylated mAb and streptavidin-HRP conjugate. Enzyme activity was quantified by tetramethylbenzidine as an indicator of the cytokine concentration in the supernatants.

## 3. Statistical Analysis

For a single comparison of 2 groups, Student's *t*-test was used. The level of significance was set at *P* = 0.05.

## 4. Results

### 4.1. CD163^+^ Macrophages and MMP-9-Bearing Cells Infiltrating in Angiosarcoma

**Figure 1:**
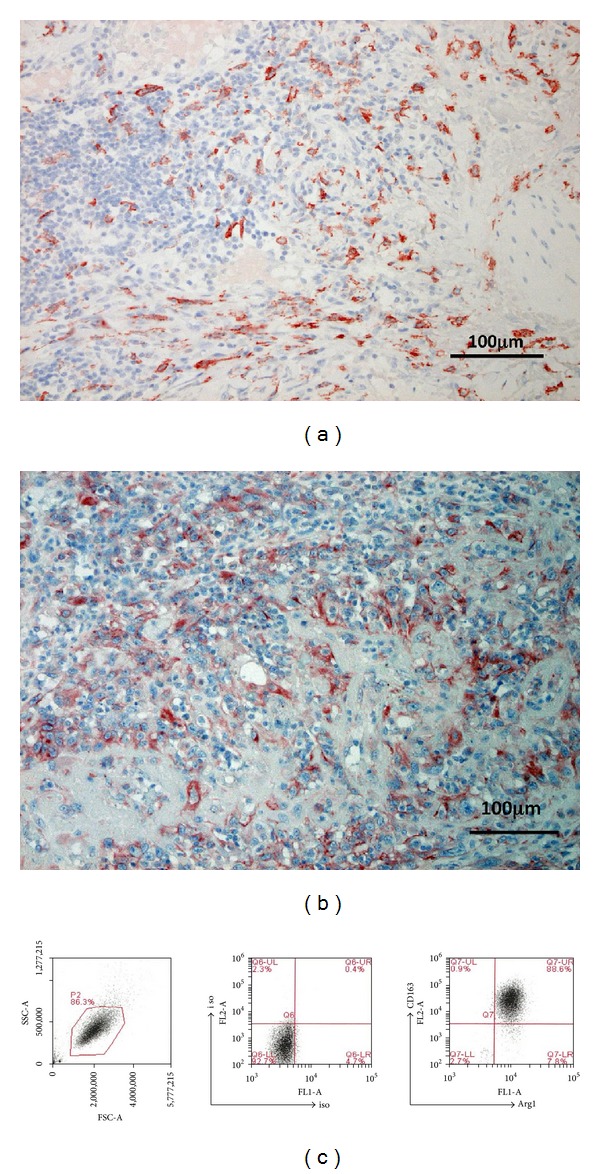
Paraffin-embedded tissue samples from patients with recurrent AS after being treated with DTX monotherapy were deparaffinized and stained as follows: anti-CD163 Ab (a) and anti-MMP9 Ab (b). Sections were developed with liquid permanent red (A) and with 3,3′-diaminobenzidine tetrahydrochloride. M2 macrophages were induced from PBMC from healthy donors as described in Material and Methods section. M2 macrophages induced from PBMC from healthy donors were cultured for 5 days, treated with human IL-4 at day 5, and stimulated with DTX and/or RS. These cells were harvested from the culture and their expression of M2 macrophage-associated molecules (CD163, Arg1) was analyzed by flow cytometry.

As we previously reported [5], immunohistochemical staining showed the infiltration of substantial numbers of CD163^+^ cells (Figure 1(a)) and MMP-9-bearing cells (Figure 1(b)) in the lesional skin of recurrent AS, which had been treated with the DTX monotherapy. 

### 4.2. The Effect of Th2 Cytokines on the mRNA Expression of CXCL10, CCL17, CCL18, IL-10, Arg1 and iNOS by M2 Macrophages

**Figure 2:**
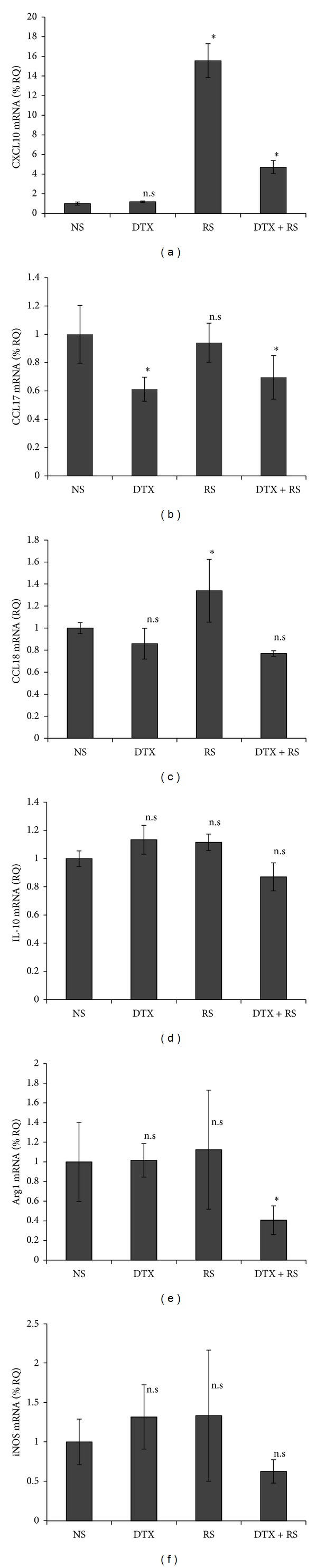
DTX with or without RS effects on the mRNA expression by M2 macrophages. M2 macrophages from PBMCs were stimulated by DTX and/or RS. Six hours after stimulation, total RNA was recovered from these cells and then reverse transcribed into cDNA. The viability of cells 6 hours after stimulation is 95%~98% in each group. Quantitative real-time PCR was conducted to determine the number of copies of cDNA for each chemokine, Arg1, and iNOS and the relative mRNA expression levels were calculated for each gene and each time point after normalization against GAPDH using the ΔΔCt method. The data from each donor were obtained by the triplicated assays, and then the mean ± SD was calculated. Representative data from three different donors are shown.

**Figure 3:**
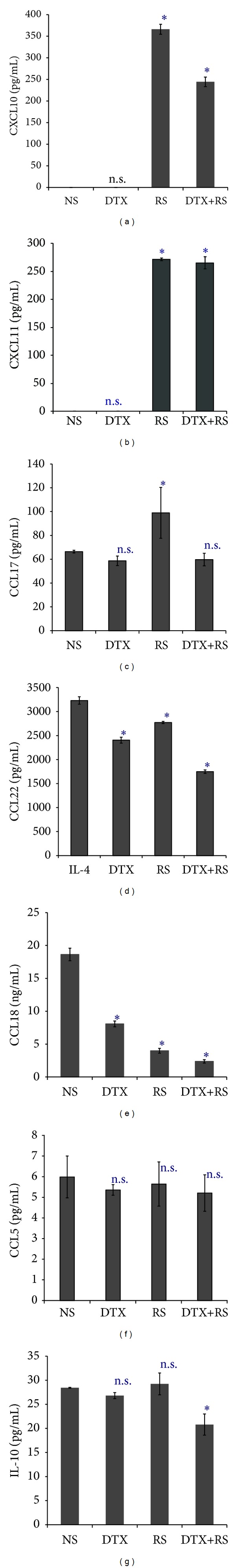
DTX with or without RS effects on the production of CXCL10, CXCL11, CCL17, CCL18, CCL22, and IL-10. The culture supernatants of M2 macrophages stimulated with DTX and/or RS were collected at 48 h after culture. Their chemokine and cytokine production was measured by ELISA. Representative data from at least three independent experiments are shown.

To examine the immunomodulatory effect of DTX and RS on M2 macrophages in AS, we generated M2 macrophages from PBMC from healthy donors as described in Material and Methods. Flow cytometry analysis revealed that these M2 macrophages were positive for CD163 and arginase 1 (Arg1), both of which were reported as markers for M2 macrophages (Figure 1(c)). In addition, these macrophages express the IL-10 mRNA and produce IL-10, which is also reported as one of the markers for M2 macrophages (Figures 2 and 3). Then, we evaluated the cytokine and chemokine mRNA expression in M2 macrophages. Among the cytokines and chemokines mRNA examined in this study, the mRNA expression of CXCL10 was significantly augmented by RS with or without DTX (Figure 2). In contrast, the expression of CCL17 and Arg1 mRNA was significantly decreased by RS with DTX (Figure 2). We did not find that these had significant effects on the expression of iNOS, and IL-10 mRNA (Figure 2). The expression of CCL18 mRNA was significantly augmented by RS alone (Figure 2).

### 4.3. The Effect of DTX and RS on the Production of CXCL10, CXCL11, CCL5, CCL17, CCL18, CCL22 and IL-10 by M2 Macrophages

We confirmed the cytokine production by M2 macrophages 48 hours after stimulation in vitro using ELISA (Figure 3). Consistent with the mRNA expression, the production of CXCL10 was significantly augmented by RS with or without DTX stimulation (Figure 3). In contrast, the production of CCL18 was significantly decreased by DTX and/or RS stimulation (Figure 3). The production of CCL22 was significantly decreased by RS with or without DTX (Figure 3). The production of CCL17 was augmented by RS (Figure 3), and the production of IL-10 was significantly decreased by RS with DTX (Figure 3). There is no significant effect of DTX and RS on the production of CCL5 (Figure 3). We could not detect the production of IFN-*γ* from each sample (data not shown).

## 5. Discussion

**Figure 4:**
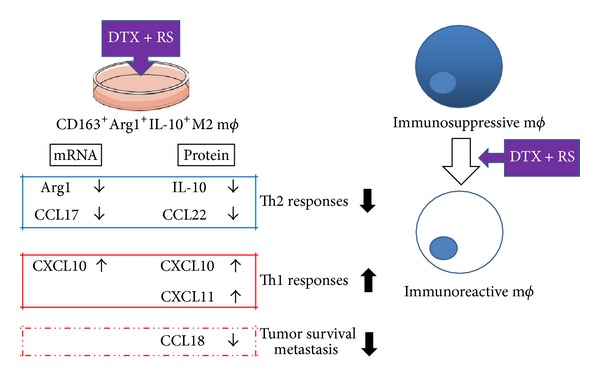
Schematic representation of immunomodulatory effect of DTX and RS together with DTX. RS modulates the chemokines and cytokines from M2 macrophages, and induces an antitumor immune response in the tumor microenvironment.

In our present report, we describe the immunomoduratory effect of docetaxel (DTX) and bisphosphonate risedronate sodium (RS) on M2 macrophages, which we previously reported as a possible supportive therapy for AS [4, 5]. Surprisingly, our present study demonstrated that DTX in combination with RS significantly upregulated the mRNA expression of CXCL10 on M2 macrophages, which was reported as a chemoattractant for the induction of CXCR3^+^ Th1 cells, effector CD8^+^ cells, NK cells, and NKT cells [7]. In contrast, DTX with RS significantly decreased the mRNA expression of CCL17 and Arg1. In addition, there was no significant effect of DTX with RS on the mRNA expression of CCL18 though RS alone significantly increased the mRNA expression of CCL18. Moreover, the production of CXCL10 and CXCL11 from M2 macrophages was significantly increased by DTX with RS though there was no effect of DTX with RS on the production of CCL17. Furthermore, DTX with RS significantly decreased the production of CCL22 and CCL18, which was previously reported to correlate with the severity and prognosis in cancer patients [8–10]. In contrast to other chemokines, there is no significant effect of DTX and RS on the production of CCL5. Our present report suggests one of the possible mechanisms of DTX with RS in the supportive therapy for angiosarcoma (Figure 4).

Like paclitaxel, DTX promotes microtubule assembly, inhibits the depolymerization of tubulin, and is reported to have higher potency [11]. However, in studies conducted in Europe, docetaxel monotherapy was rather ineffective for soft tissue sarcoma, including angiosarcoma, and could not be recommended for further use [11]. Therefore, further supportive reagents are necessary for the treatment of AS. The use of bisphosphonates (BPs) in malignancy has been increasing [12, 13]. Recent reports suggested that BPs might have a direct antitumoric effect beyond their beneficial effect on bone metastasis [12]. Various investigations have demonstrated a synergistic, antiproliferation effect of BPs with conventional chemotherapeutic drugs in vitro [14, 15]. Indeed, Fehm et al. reported that the antitumor effect of BPs for breast cancer cells in vitro was equal or even superior to those of chemotherapeutic drugs, such as DTX [15]. In addition, from the immunological point of view, it was reported that pharmacological inhibition of MMP9 by aminobisphosphonate decreased pro-MMP9 and VEGF in the serum and abrogated the induction of immunosuppressive macrophages, myeloid derived suppressor cells (MDSCs), in the tumor microenvironment [16].

From the above reports, we hypothesized that RS has a synergistic effect with DTX in the anti-tumor immune response, especially in targeting immunosuppressive M2 macrophages. As a previous report suggested, DTX polarized MDSCs towards M1-like functional macrophages in tumor-bearing hosts, suggesting possible immonomodulatory effects of DTX on MDSCs [17]. In our present study, DTX decrease the expression of CCL17 mRNA and production of CCL18 from M2 macrophages but did not significantly affect the expression of CXCL10, CCL18 and Arg1 mRNA expression, nor the production of CXCL10, CCL17 and IL-10 from M2 macrophages. Interestingly, DTX in combination with RS significantly augmented the expression of CXCL10 mRNA expression and decreased the expression of CCL17 and Arg1 mRNA expression on M2 macrophages. Furthermore, in parallel with the mRNA expression, the production of CXCL10 was significantly augmented, and the production of IL-10 was significantly decreased by DTX with RS. In addition, the production of another Th1 chemokine, CXCL11 was significantly augmented and another Th2 chemokine, CCL22, was significantly decreased by DTX with RS. In contrast to the mRNA expression, the production of CCL18 was significantly decreased by DTX with RS. This discrepancy might be explained by regulatory processes occurring after mRNA is made as Vogel and Marcotte reported [18]. In aggregate, our present data suggested that the therapeutic effect of DTX with RS on the cutaneous angiosarcoma might correlate with the induction of immunoreactive CXCR3^+^ Th1 cells, effector CD8^+^ cells, NK cells, and NKT cells by the increased levels of CXCL10 and CXCL11 production from these modified macrophages. In addition, as previous reports suggested, the production of CCL18 is correlated with the severities of several malignant tumors [8–10], and one of the potential producers of CCL18 is CD163^+^ macrophages [10]. The decreased levels of CCL18 from M2 macrophages might correlate with the therapeutic effects of DTX with RS in patients with angiosarcoma [5]. Our present report suggests one of the possible mechanisms of DTX with RS in the supportive therapy for AS.
